# Calcitonin gene relating peptide inhibitors in combination for migraine treatment: A mini-review

**DOI:** 10.3389/fpain.2023.1130239

**Published:** 2023-03-17

**Authors:** Tulsi Shah, Kate Bedrin, Amanda Tinsley

**Affiliations:** Department of Neurology, MedStar Georgetown University Hospital, Washington, DC, United States

**Keywords:** migraine, chronic migraine, CGRP monoclonal antibodies, CGRP receptor antagonist, CGRP inhibitor, migraine therapy, refractory migraine

## Abstract

The discovery of calcitonin gene-related peptide (CGRP) and its role in migraine pathophysiology has led to advances in the treatment of migraine. Since 2018, the Food and Drug Administration (FDA) has approved four monoclonal antibody (mab) therapies targeting either the CGRP ligand or receptor and 3 oral small molecule CGRP receptor antagonists. These targeted therapies have been shown to be safe and effective for either preventive or acute treatment of migraine in adults. Given their efficacy and tolerability profile, CGRP inhibitors have revolutionized the approach to migraine treatment. Theoretically, combining therapies within this therapeutic class could lead to more CGRP blockade and, subsequently, improved patient outcomes. There are providers currently combining CGRP therapies in clinical practice. However, limited data are available regarding the efficacy and safety of this practice. This mini-review provides a summary of available data and poses important considerations when combining CGRP therapies for migraine treatment.

## Introduction

CGRP is a neuropeptide involved in migraine pathophysiology and is a target for both acute and preventive migraine treatment (1). There are currently four CGRP mabs that are used for migraine prevention: erenumab 70 mg or 140 mg subcutaneously (SC) monthly, fremanezumab 225 mg SC monthly and 675 mg SC quarterly, galcanezumab 240 mg SC loading dose followed by 120 mg SC monthly and eptinezumab 100 mg or 300 mg intravenously (I.V.) quarterly (2–5). There are three available oral small molecule CGRP receptor antagonists—ubrogepant 50 mg and 100 mg orally (po) for abortive therapy, atogepant 10, 30, and 60 mg po daily for migraine prevention, and rimegepant 75 mg orally disintegrating tablet (ODT) as needed every 24 h for abortive therapy or 75 mg every other day scheduled for migraine prevention (6–9). Questions arise regarding the utility and safety of combining treatments resulting in dual CGRP blockade. This article addresses the current data available and areas where further research is needed to enhance our understanding of combining treatments targeting CGRP.

## Background on CGRP and mechanism of action of CGRP inhibitors

CGRP is a vasoactive neuropeptide involved in cerebrovascular regulation that is expressed throughout the trigeminovascular system and plays a major role in migraine pathophysiology (10, 11). CGRP is a potent vasodilator of cerebral blood vessels (12). During a migraine attack, CGRP is released from the trigeminovascular system following trigeminal nerve activation (13). CGRP release induces neuroinflammation and leads to peripheral and central sensitization (14). The involvement of CGRP in migraine is evidenced by elevated serum CGRP levels in the external jugular vein during a migraine attack (15). I.V. Infusion of CGRP resulted in a migraine attack in individuals with a history of migraine (16). Plasma CGRP levels decreased in parallel with headache intensity following administration of a triptan, a first-line migraine-specific acute treatment (17, 18). Given the role of CGRP in migraine pathophysiology, multiple therapies have been designed with the goal of blocking the CGRP-signaling pathway.

CGRP mabs are effective and well-tolerated preventive migraine therapies. Erenumab is the only mab that directly blocks access of ligands to the CGRP receptor. Although it has high affinity and selectivity for the receptor (2, 19), it also has been shown to antagonize the amylin (AMY_1_) receptor. This further demonstrates the complexity of the CGRP receptor and calcitonin family (20). Eptinezumab, fremanezumab, and galcanezumab bind the CGRP ligand rather than the receptor (3–5). All four available CGRP mabs are metabolized by the reticuloendothelial system, degraded by enzymatic proteolysis into small peptides and amino acids with erenumab eliminated mainly by saturable binding to the target CGRP receptor at low concentrations (21).

Small molecule CGRP receptor antagonists, so-called gepants, are effective for both preventive and acute migraine treatment. Preclinical studies showed evidence that CGRP receptor antagonists blocked photophobia in a mouse model (22). Spinal trigeminal nucleus activity in response to activation of the trigeminal nociceptive system is inhibited *via* pre-treatment with a CGRP receptor antagonist (23). Additional preclinical research showed inhibition of CGRP and trigeminovascular-induced vasodilation *via* CGRP receptor antagonists (24, 25). These data were used to support further development of the gepants. Ubrogepant and Rimegepant have been shown to be effective and safe acute migraine treatments (6, 26). Rimegepant and atogepant are available as preventive migraine therapies (8, 9). The gepants are metabolized mainly *via* pathways in the liver and to a lesser extent, kidneys (accessdata.fda.gov, reference IDs: 4864125, 4538691, and 4802639) (27–30). Of note, liver enzyme elevation was observed with first generation gepants.

Gepants and erenumab act on CGRP receptors, while the other CGRP mabs bind the CGRP ligand. Combining a gepant with a CGRP mab other than erenumab leads to CGP antagonism in two different parts of the pathway—receptor and ligand. Dual-blockade of the CGRP receptor itself may have certain benefits. The fact that gepants cross the blood brain barrier may also add to a synergistic effect with a CGRP mabs. More research is needed to better understand the benefit and consequence of combining gepants with certain CGRP mabs based on their different roles in the CGRP pathway.

In addition to the nervous system, CGRP is found in the gastrointestinal, cardiovascular, endocrine, renal, skin, and immune systems. Increased CGRP blockade could potentially lead to off-target effects in these other systems. Constipation has been reported as an off-target effect with gepants and CGRP mabs [(21), accessdata.fda.gov reference ID: 4264882; accessdata.fda.gov reference ID: 4864125]. However, in clinical trials CGRP modifying treatments were typically well tolerated overall. Further real-world data is needed to better understand long-term consequences of CGRP blockade (1). CGRP may have a protective role in the cardiovascular and cerebrovascular systems through vasodilation. It is thought to protect against myocardial infarction and heart failure following cardiac ischemic and is also thought to protect against focal cerebral ischemia through vasodilation resulting in increased cerebral blood flow (31). More research is needed to better understand the consequences of long-term CGRP blockade on the vascular system and compensatory mechanisms. Further research is also needed to identify the vascular and compensatory ramifications of dual-blockade of CGRP when an individual is using more than one CGRP-targeted treatment. This need for further research can be extrapolated to all systemic systems influenced by CGRP as it is a complex neuropeptide which functions throughout the body. Theoretically, long-term dual blockade of CGRP may have an impact on these various functions, potentially increasing the likelihood of adverse effects, such as ischemia and constipation.

## Combining CGRP monoclonal antibodies for preventive treatment with gepants for acute treatment of migraine

In clinical practice, there are headache specialists that are combining CGRP mabs for preventive treatment with gepants used for the acute treatment of migraine. There have been several case studies that have explored this combination specifically.

One case report reviewed two patients with nearly twenty years of refractory migraine and suboptimal response with other previously tried migraine medications. The first patient was given rimegepant 75 mg ODT as needed, up to once daily, for treatment of acute attacks. Within one week of use, she had substantial relief and successfully treated 7 out of 7 acute attacks with rimegepant only and was able to eliminate use of her typical ibuprofen and a caffeinated analgesic. After 6 months of therapy with rimegepant, she continued to have frequent migraine attacks and was thus started on erenumab 70 mg SC monthly as a preventive therapy, which reduced her monthly migraine days from 13 to 7 within the first month. She continued to use rimegepant for acute migraine management with significant improvement, illustrating efficacy of combining these two treatment methods. The second patient had a baseline of 22 migraine attacks per month and was subsequently started on rimegepant 75 mg as needed, up to once daily, for treatment of acute attacks. She had significant benefit of using rimegepant for treating acute migraine attacks (16 attacks in the first month and 11 attacks in the second month). She was also able to stop her other medications in month 2, including ondansetron, ketorolac, and diphenhydramine. Given her continued high migraine attack frequency, she was started on erenumab 140 mg SC monthly after the second month. She experienced 9 migraine attacks within the first month of using erenumab, all of which responded to rimegepant as an abortive agent. Both patients experienced no adverse events on this combination (32, 33).

A larger scale multicenter, open-label, long-term safety study reviewed 13 patients with migraine who were concurrently treated with one of the CGRP mabs for preventive therapy and rimegepant for acute therapy. Of the 13 patients, 7 were being treated with erenumab, 4 with fremanezumab, and 2 with galcanezumab. These patients were treated with rimegepant as needed for the acute treatment of migraine, with the mean treatment period being 9.6 weeks and mean exposure of rimegepant within a 4-week period being 7.8 doses. Five patients in this study reported an adverse effect considered mild to moderate in severity. Nasopharyngitis was the most common adverse event affecting 2 out of 13 patients. The other adverse events reported affecting single patients were back pain, myalgia, contusion, dizziness, sinusitis, first-degree AV block, and viral gastroenteritis. However, no patients had serious adverse effects or adverse effects significant enough to discontinue combination therapy. Thus, this study suggests that rimegepant as an oral acute treatment may be safe to use concurrently with CGRP mabs used as preventive treatment (32, 34).

An open label longitudinal treatment study compared adverse events of CGRP receptor antagonists combined with CGRP mabs to CGRP receptors antagonists alone or with other standard of care preventive medications. There was no significant difference in adverse events in the group using combined CGRP mabs for prevention and gepants for acute treatment of migraine (35). This study further supports the idea that coadministration of CGRP targeted treatments may be safe for a subset of patients with migraine.

One multicenter, open-label, phase 1b trial evaluated the pharmacokinetics and safety of combining either erenumab or galcanezumab with ubrogepant. 40 patients were randomized to either ubrogepant with erenumab or ubrogepant with galcanezumab. The study reviewed plasma ubrogepant concentration in relation to administration of the mab and found no significant difference in the pharmacokinetic profile and no safety concerns with coadministration (36).

## Combining CGRP monoclonal antibodies for preventive treatment with gepants for preventive treatment of migraine

Although the aforementioned studies (32–34) have addressed migraine patients taking both CGRP mabs for prevention with gepants for abortive treatment, there has not yet been to our knowledge a study that has reviewed combination of CGRP mabs with gepants specifically for preventive treatment. Given that CGRP receptor antagonists cross the blood-brain barrier (37) whereas CGRP mabs do not, dual blockade of CGRP centrally may lower free CGRP levels even further resulting in more symptomatic relief. However, this may also suggest the plausibility of more adverse effects and decreased tolerability in patients. Further studies are needed to better assess the efficacy, safety, and tolerability of combing these two migraine preventive strategies.

## Combining gepants for preventive treatment with gepants for acute treatment of migraine

Similarly, the efficacy and safety of using more than one gepant simultaneously for prevention and acute management in migraine has not yet been systematically studied to our knowledge. Rimegepant has been shown to be safe when taken up to 18 days monthly and has been approved to be used as prevention on a scheduled basis (every other day) with the option to dose as needed for acute therapy if not taken that day for prevention (9, 38). There is a study planned to assess efficacy and safety of combining daily atogepant for migraine prevention with ubrogepant as needed for acute therapy (clinicaltrials.gov identifier: NCT05264129). Further research is needed to better our understanding on safety, tolerability, and efficacy of combining two separate gepants for prevention and acute migraine therapy.

## Discussion

Treatment options for migraine should be tailored to the individual patient, and combination of CGRP inhibitors may be considered if appropriate. Both gepants and CGRP mabs have been shown to be effective when used individually; however, this combination may offer a synergistic benefit in individuals that experience a suboptimal response to either CGRP agent alone or other standard therapies. Given that gepants cross the blood-brain barrier whereas CGRP mabs act peripherally, it is reasonable to consider that the combination may have a synergistic effect that may lead to improved efficacy compared to either therapy alone (37). Furthermore, a synergistic effect may exist due to differences in metabolism, with CGRP mabs metabolized *via* the reticuloendothelial system and gepants metabolized *via* hepatic and renal pathways.

It has been well established that free CGRP levels are higher during acute migraine attacks and that there is a correlation in reduction of CGRP levels with migraine relief (40). Mabs targeting CGRP itself, i.e., the ligand, work by binding of the antibody and ligand resulting in reduced free ligand available for the receptor. The efficacy of this is improved with a longer duration in reduction of free ligand concentration (41). A review of the pharmacokinetics of galcanezumab suggested that free CGRP ligand levels are reduced in a time and dose dependent manner. This analysis showed that the average steady state decrease in the concentration of free CGRP of galcanezumab at 120 mg monthly was 61% and 240 mg was 76%, though of note, these concentrations did vary from initial time of administration to the end of the month. Additionally, the CGRP ligand levels were measured in the serum and not within the trigeminal tissues where we believe CGRP is having the biggest impact on migraine pathophysiology (41). Another study showed the presence of up to 55% free CGRP concentration even with mab treatment, which implicates that combination therapy with a CGRP receptor antagonist or another mab may have an additive effect (42).

Furthermore, another factor to consider is different therapeutic benefits due to the varying pharmacokinetics of CGRP agents. Gepants and CGRP mabs have different routes of administration, with the former being administered parenterally and the latter orally. Because of this, their peak concentrations differ, which plays a role in efficacy, free CGRP levels, and potential side effects. Taking pharmacokinetics into consideration, it is unlikely that CGRP mab and gepants would have significant drug to drug interactions, although further study is warranted to confirm this. A randomized phase 1b study showed no significant change in pharmacokinetics or adverse effects of ubrogepant with coadministration of either erenumab or galcanezumab compared to either of these medications alone (36).

Since CGRP plays a role in regulation of multiple organ systems and homeostasis, the concern exists that combining two CGRP antagonists may adversely affect physiological CGRP and be harmful (32). CGRP is a potent vasodilator, affecting the cerebral, coronary, and renal vasculatures. Its vasodilatory effects cause systemic regulation of blood pressure and affects the cardiovascular system and healing of wounds (43), playing a protective role in hypertension *via* smooth muscle cell vasodilation in the vascular walls (31). Thus, there is speculation that combining two CGRP inhibitors may result in hypertension and increased risk of ischemic events (32). There is some clinical data that suggests an increased incidence of hypertension associated with the use of several CGRP mabs when used individually, namely erenumab and fremanezumab. One study assessed 211 patients who were started on a CGRP agent, 109 with erenumab and 87 with fremanezumab. The results showed that 47.7% of patients in the erenumab group and 27.6% in the fremanezumab group had a systolic blood pressure rise of ≥20 mm Hg and/or a diastolic BP rise ≥10 mm Hg at any point during course of treatment. It should be noted that increasing the dosage of erenumab from 70 to 140 mg did not change blood pressure further. Additionally, 3.7% of patients treated with erenumab were diagnosed with new onset hypertension requiring treatment. In general, this study suggests that erenumab has a more consistent effect on increase in blood pressure than fremanezumab; however, this does show that CGRP mabs may result in increases in blood pressure and potentially new onset hypertension in a subset of patients (44).

Animal studies that tested antibodies against CGRP showed mucosal damage in the gastrointestinal tract and given that CGRP modulates gastrointestinal motility, it is possible that CGRP blockade may be related to GI side effects such as constipation (45). A common adverse reaction in clinical trials of erenumab and atogepant was constipation, with an incidence of 3% for erenumab (accessdata.fda.gov reference ID: 4264882) and 6% for atogepant (accessdata.fda.gov reference ID: 4864125).

When taking these adverse effects into consideration, it is important for prescribers to discuss potential adverse effects with patients, especially patients with pre-existing gastrointestinal motility issues. Additionally, dual CGRP blockade may not be the best option in patients with pre-existing hypertension or significant cardiovascular risk factors, though of note, there is currently no data that suggests hypertension as a side effect of gepants and thus combining mabs with gepants may still be a safe option. There is currently no direct evidence that combining two CGRP inhibitors can result in hypertension and increased risk of cardiovascular events, though it must be noted that the adverse effects mentioned may be a limiting factor when considering dual CGRP blockade. More research is needed to better understand the safety profile of combining various CGRP inhibitors.

Most of the data for combining CGRP inhibitors is regarding the combination of CGRP mabs for migraine prevention with gepants for acute migraine treatment. In this mini-review, several smaller scale studies were reviewed that investigated the efficacy and safety profile of combining CGRP agents, with sample sizes ranging from 2 to 40 individuals. While data is limited, the combination of CGRP mabs for migraine prevention with gepants for acute migraine treatment may provide benefit in patients with treatment refractory migraine. No significant adverse effects were reported in the available studies.

Combining CGRP inhibitors for migraine treatment is a controversial topic with many research gaps. First, there have not been any highly powered randomized trials regarding coadministration of these agents. Second, there are no studies that have reviewed combining a gepant for preventive management with a CGRP mab for prevention or studies that have reviewed combining a gepant for preventive management with a gepant for abortive management (other than rimegepant being used as dual therapy with favorable safety profiles reported with use up to 18 days/monthly). Further research is needed to evaluate the safety, tolerability, and efficacy of combining CGRP-targeting treatments and the resulting dual CGRP blockade. Nonetheless, preliminary studies of combining CGRP inhibitors suggest this topic warrants further investigation, as the combining of treatments may be beneficial especially for individuals with suboptimal migraine treatment regimens.
